# What Is the Appropriate Acupuncture Treatment Schedule for Chronic Pain? Review and Analysis of Randomized Controlled Trials

**DOI:** 10.1155/2019/5281039

**Published:** 2019-06-18

**Authors:** Yan-Jiao Chen, Cai-Tao Chen, Jia-Yuan Liu, Gabriel Shimizu Bassi, Yong-Qing Yang

**Affiliations:** Shanghai Research Institute of Acupuncture and Meridian, Shanghai University of Traditional Chinese Medicine, 650 South Wanping Road, Shanghai 200030, China

## Abstract

**Background:**

Acupuncture is widely used for the treatment of chronic pain. Different protocols of acupuncture practice exist and lack agreement on the optimal schedule of acupuncture treatment.

**Objective:**

To review the appropriate acupuncture treatment schedule for chronic pain.

**Methods:**

Embase, Pubmed, Cochrane Central Register of Controlled Trials, and reference lists were searched from 2009 to 2018 to identify randomized controlled trials of acupuncture for chronic pain conditions. We collected factors of treatment schedule (D, duration of each treatment session; N, number of treatment sessions; T, total duration of treatment in weeks) from each of the trials, and the linear regression analysis with real pain relief rate (both treatment and follow-up) was performed. Furthermore, we recommend the concept of “DOSE” and frequency (F) to evaluate the dose and frequency effect of acupuncture.

**Results:**

Twenty-four trials with a total number of 3461 patients met the inclusion criteria. Of these, data from 23 studies were available for analysis. Firstly, the results showed that follow-up pain relief rate was decreased slightly with the increase of the duration of each session and DOSE (r=-0.3414 and r=-0.3246, respectively), but those two factors had no correlation with the pain relief rate after treatment. Secondly, it showed that either lower frequency with 2 sessions/week and higher frequency greater than 2 sessions/week or DOSE of 30 mins/week can achieve higher pain relief rate after treatment. Thirdly, we found the rate of pain relief remained at a high level greater than 20% up to 18 weeks after the treatment, and then it dropped sharply below 10% with the follow-up extended. A positive relationship was found between study score and pain relief both in treatment and follow-up (r=0.4654 and r=0.3046, respectively).

**Conclusions:**

The effect of acupuncture varies greatly with the different schedules of acupuncture, so it is necessary to review and choose the appropriate schedule. Although the current work is based on a limited number of trials, the findings suggest that acupuncture has a dose and frequency effect presenting within a certain range, which would have considerable implications for the design and interpretation of clinical trials. More high-quality randomized controlled trials on acupuncture schedule research were needed for providing more definitive evidence.

## 1. Introduction

Acupuncture has been used for more than 2500 years in China [1]. The World Health Organization (WHO) listed pain and other 42 indications for acupuncture in 1979 [2] and 107 diseases or disorders in 2002 [3]. A recent study found pain represents the largest category among the top 99 indications in private clinics in the United States [4]. Meanwhile, the National Academies of Sciences, Engineering, and Medicine (NASEM) pointed out that acupuncture has become common practice for pain relief in recent decades [5]. Acupuncture is now widely used as a powerful tool for controlling pain conditions.

In many of the pain conditions where acupuncture is used, there is limited high-quality evidence to draw clear conclusions over its effectiveness [6, 7]. Researchers argue that the efficacy of acupuncture is difficult to be confirmed in clinical trials because a reasonable effective control group was used [8]. More researchers are studying how sham acupuncture, the most commonly used control in acupuncture research, should be operated to avoid producing specific effects of acupuncture resulting from blunt needles [9], needled outside known points [10], and acupoints not indicated for conditions [11]. However, those sham acupuncture methods should not be used by acupuncture proponents to explain negative results unless when they are supported by evidence.

Other than the control methods, there are many other factors affecting the efficacy of acupuncture, one of which is that the treatment protocol cannot be unified. Different clinical trials used different protocols and a protocol used by an acupuncturist might be dismissed by another [12, 13]. In 2010, the revised STandards for Reporting Interventions in Clinical Trials of Acupuncture (STRICTA) were published to encourage the publication protocol of precise interventions methods used in trials of acupuncture [14]. The recommendations emphasized the need to provide rationed detailed protocol information, such as needling, treatment regimen, cointerventions, practitioners background, and control interventions. However, suggested* schedule*, the key component of protocol, including the range of duration of each treatment session, number of treatment sessions, and total duration of treatment in weeks for diseases, is not given.

The effect of factors of* schedule* on acupuncture was ignored for a long time. In the present study, a review of acupuncture randomized controlled trials for the treatment of chronic pain conditions was conducted from 2009 to 2018. In addition, schedule related factors influencing pain relief during both acupuncture treatment and follow-up period were investigated.

## 2. Methods

### 2.1. Selection and Exclusion Criteria

To be included, studies met the following criteria: (1) randomized controlled trials; (2) pain-related problems (for example, knee osteoarthritis, low back pain, fibromyalgia, shoulder pain, subacromial impingement, neck pain, myofascial pain, pelvic pain, headache, and plantar fasciitis); (3) patients ≥ 18 years old; (4) classical acupuncture intervention (only needles); (5) chronic pain (pain lasting more than 3 months).

Excluding criteria are as follows: (1) healthy volunteers and pregnancy; (2) cancer and menstrual pain; (3) auricular, dry needling, tongue, microsystems, intradermal, laser, acupressure, apipuncture, scalp, facial, and electrical acupuncture.

#### 2.1.1. Type of Controls

Inclusion criteria are as follows: (1) no treatment or waiting list; (2) usual care (including medicine therapy); (3) physiotherapy; (4) relaxation; (5) self-educational programs; (6) manipulation; (7) superficial acupuncture; (8) nonpenetrating needles; (9) insertion simulation at nonacupoints/acupoints away; (10) application of placebo TENS or laser; (11) exercise.

Exclusion criteria are as follows: (1) no control; (2) same acupuncture with different number of needles/sessions.

### 2.2. Data Sources and Searches

We searched Embase database, Pubmed database, and the Cochrane Central Register of Controlled Trials from 2009 to 2018. Our search strategy was selected by the iteration of the words “chronic pain”, “acupuncture”, etc. (detailed retrieval type was provided in Supplementary File 1). Chinese trials and trials from Chinese databases were not considered. Trials were firstly selected by screening the titles and abstracts of all references and assessed by two reviewers. If included in the study, the article is fully checked. Any disagreement was solved by discussion and reanalysis of the data.

### 2.3. Data Extraction and Quality Assessment

The following aspects were considered: N, number of treatment sessions; D, duration of each treatment session; T, total duration of treatment in weeks; results on the pain outcome measures; follow-up pain outcomes; and supplement data. A second reviewer checked all extracted trials results against the original publications.

For the pain evaluation scale, we used the following priority order: Visual Analogue Scale (VAS), Western Ontario and McMaster Universities Osteoarthritis (WOMAC), Northwick Park Questionnaire (NPQ), Short Form Health Survey (SF-36), Shoulder Pain and Disability Index (SPADI), National Institutes of Health Chronic Prostatitis Symptom Index (NIH-CPSI), Symptom Bothersomeness Scale, and Knee Society Score (KSS). In addition, the following strategy for pain outcome was used: (1) If the pain-related scale was a comprehensive scale, the part about the pain measurement was selected (for example, in WOMAC, we chose the part of mean WOMAC pain). (2) Both treatment pain relief score and follow-up pain relief score should be as close as possible to the completion of the treatment and follow-up. (3) Missing data were acquired by contacting the original study authors. If the missing data cannot be obtained, we analyzed the available data (disagreements were solved by discussion). (4) When meeting the interval value parameters, mean values were used.

#### 2.3.1. Quality Assessment

Two reviewers independently evaluated the methodological quality of the included studies using the combination of Cochrane risk of bias tool [15] and the completeness of the STandards for Reporting Interventions in Clinical Trials of Acupuncture (STRICTA) checklist. Any disagreement was solved by discussion and reanalysis of the data.

### 2.4. Data Synthesis and Analysis

Statistical significance of differences between the results was tested using the one-way ANOVA. Linear regression was used to model the relationship between two variables. Results were considered significant when P < 0.1. This review is reported in accord with Preferred Reporting Items for Systematic Reviews and Meta-Analyses (PRISMA).

#### 2.4.1. Measures of Treatment Effect

(1) Pain relief rate was calculated based on the following formula.

(1) $$\begin{array}{c} Pain\;\;relief\;\;rate=\left|\;\frac{Mean\;\;Pain\;\left(treatment/follow\text{-}up\right)-Mean\;\;Pain\;\left(baseline\right)}{Mean\;\;Pain\;\left(baseline\right)}\times 100\%\;\right| \end{array}$$

(2) Real pain relief rate was calculated based on the following formula.

(2) Real  pain  relief  rate=Pain  relief  rateacupuncture−Pain  relief  ratecontrol

#### 2.4.2. Assessment of Treatment Schedule

(1) The concept of “DOSE” for measuring the dose of acupuncture was defined, which was calculated based on the following formula: DOSE = D×N/T (D, duration of each treatment session; N, number of treatment sessions; T, total duration of treatment in weeks).

(2) We used a similar concept for calculating the frequency of treatment based on the following formula: F= N/T (F, frequency; N, number of treatment sessions; T, total duration of treatment in weeks).

## 3. Results 

### 3.1. Literature Search and Description of Included Studies

**Figure 1:**
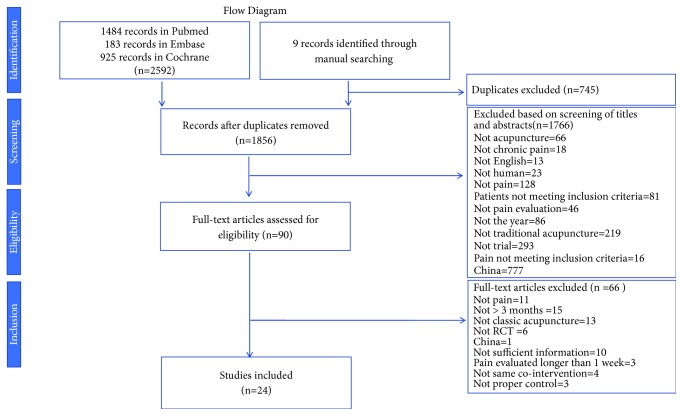
Flow diagram of trials selection process.

Literature search identified 2592 references by using search method and 9 others were further added by manual search. 745 duplicates were excluded and 1766 studies were removed by screening. 90 studies were assessed and further analysis excluded 66 records; a total of 24 articles were included finally. The process of study selection was described in Figure 1.

In twenty-four trials, a total of 3461 patients (median 64, minimum 16, and maximum 638) were assessed (Table 1). Four trials addressed headache (737 patients) and fibromyalgia (319 patients); three trials addressed low back pain (807 patients), headache (237 patients), and knee osteoarthritis (551 patients); two addressed neck pain (556 patients) and jaw pain (44 patients); and one addressed arm pain (50 patients), myofascial pain (60 patients), and pelvis and hip pain (100 patients). Nine studies set more than two controls, and six clinical trials lacked follow-up period.

### 3.2. Study Quality

**Figure 2:**
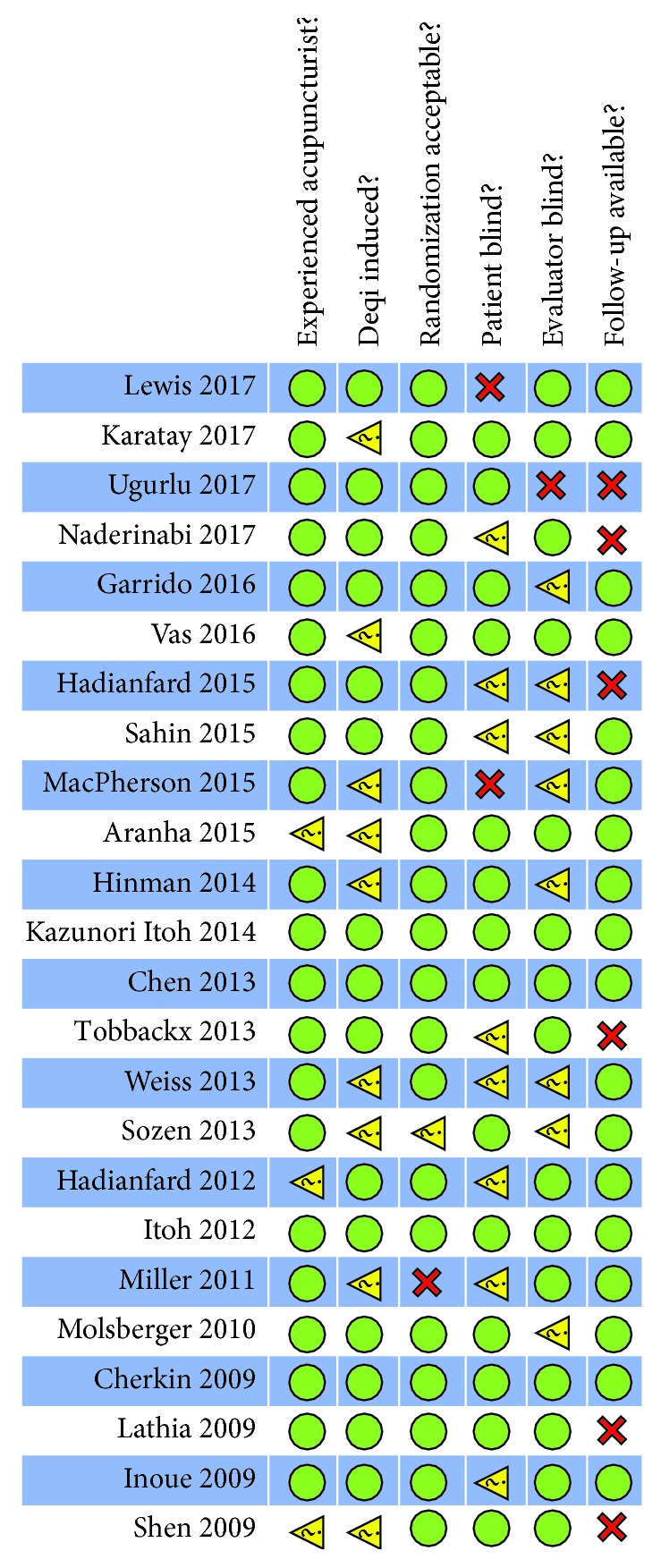
Risk of bias summary.

The risk of bias of each included study as a checklist of quality is presented in Figure 2. Twenty-two trials specified the method of randomization, while one study stated that participants were randomized to groups but failed to provide a more detailed description [16], and one used a very simple allocation by randomly selected pieces of paper [17]. Twenty-one studies very well provided the experiences of acupuncturist, and three did not [18–20]. Nine studies do not mention the requirements of “deqi” sensation [13, 16–18, 20–24]. Although it was not feasible to blind the participants when sham controlled efficacy trials were conducted, eight studies failed to provide the information about blinding [17, 19, 24–29] and two trials revealed that they did not perform blinding [23, 30]. Eight trials did not report the blindness of outcome evaluators [16, 22–24, 27, 29, 31, 32], and one study revealed that they did not have blind evaluators [12]. Six trials did not conduct the follow-up research [12, 20, 26, 28, 29, 33]. Additionally, one of these trials failed to report baseline SPADI information [33].

### 3.3. Outcome Measurements

#### 3.3.1. Assessment of the Linear Regression Model between Real Treatment Pain Relief Rate and Schedule Related Factors

**Figure 3:**
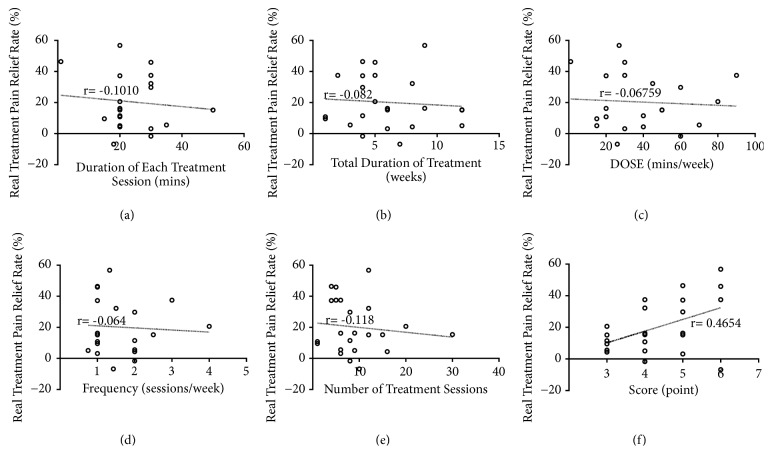
Correlation coefficients between real treatment pain relief rate and the following factors: (a) duration of each treatment session; (b) total duration of treatment; (c) DOSE; (d) frequency; (e) number of treatment sessions; (f) study quality score; the higher the score, the better the quality of the study.

The correlation coefficients between each schedule related factor (sample size, duration of each treatment session, total duration of treatment, number of treatment sessions, frequency, DOSE and score) and real treatment pain relief rate were analyzed as shown in Figure 3 and Table 2. Data showed that there was almost no liner relationship between each of other factors and real treatment pain relief rate, Figures 3(a)–3(e). In Figure 3(f), a positive correlation (r=0.4654) was found between study quality score and pain relief rate after treatment. It indicated that study quality was associated with the size of acupuncture effect.

#### 3.3.2. Assessment of the Linear Regression Model between Real Follow-up Pain Relief Rate and Schedule Related Factors

**Figure 4:**
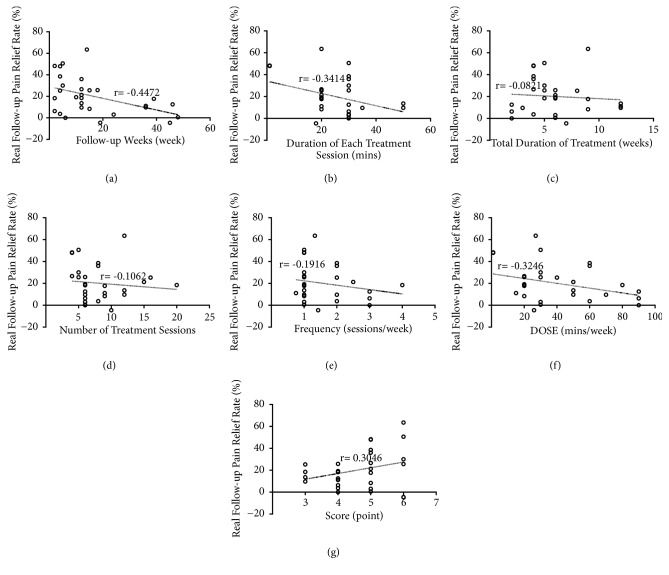
Correlation coefficients between real follow-up pain relief rate and the following factors: (a) follow-up weeks; (b) duration of each treatment session; (c) total duration of treatment; (d) DOSE; (e) frequency; (f) number of treatment sessions; (g) study quality score; the higher the score, the better the quality of the study.

The results of the linear regression analysis were presented in Figure 4 and Table 3. From Figure 4(a), the result showed that the real follow-up pain relief rate declined with the extending of follow-up period.

In addition, the real follow-up pain relief rate was decreased with the increase of the duration of each treatment session and DOSE in Figures 4(b) and 4(f). Either shorting the treatment time of each session or decreasing the DOSE of treatment per week can achieve better treatment outcome. Meanwhile, it was noted that the real follow-up pain relief rate presented a positive correlation (r=0.3046) with study quality score (Figure 4(g)), which was the same with the real pain relief rate after treatment. The effect of the score on pain relief rate was maintained to the follow-up period.

#### 3.3.3. Evaluation of the Schedule Related Factors That Affect Real Pain Relief Rate

**Figure 5:**
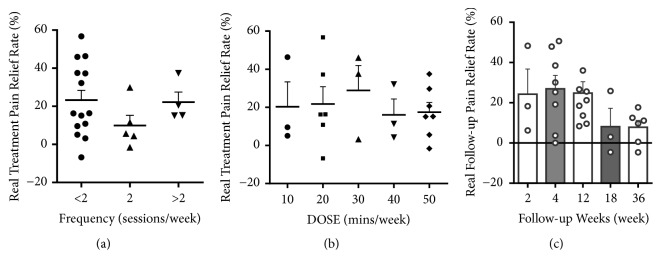
Relationship between acupuncture schedule related factors and pain relief rate. (a) The relationship between frequency and real treatment pain relief rate: frequency<2, including 3 sessions/4 weeks, 1 session/week, 4 sessions/3 weeks, 10 sessions/7 weeks, and 3 sessions/2 weeks; frequency=2, including 2 sessions/week; frequency>2, including 5 sessions/2 weeks, 3 sessions/week, and 4 sessions/week. (b) The relationship between DOSE and real treatment pain relief rate: DOSE was divided by cumulative stimulus time. (c) The relationship between follow-up weeks and real follow-up pain relief rate: follow-up weeks were divided by the length of weeks.

In order to determine the appropriate frequency and DOSE of acupuncture treatment, each parameter was divided into several parts and one-way ANOVA analysis was carried out as shown in Figures 5(a) and 5(b). As we can see, lower frequency with 2 sessions/week and higher frequency greater than 2 sessions/week can achieve higher real treatment pain relief, while the one with 2 sessions/week can lead to lower pain relief, although there was no significant statistical difference among groups.

In Figure 5(b), the higher DOSE of acupuncture did not guarantee better treatment pain relief. The rate of pain relief increased slightly as the DOSE of acupuncture increased, peaked at the DOSE of 30 mins/week, and then declined steadily along the extending of DOSE.

Furthermore, we separated follow-up weeks into 5 time points, and the pain relief effect of each time was analyzed in Figure 5(c). Up to the 18 weeks of follow-up, the rate of pain relief remained at a high level greater than 20% but it dropped sharply below 10% after 18 weeks. Thus, 18 weeks might be the expiry date of acupuncture treatment.

**Figure 6:**
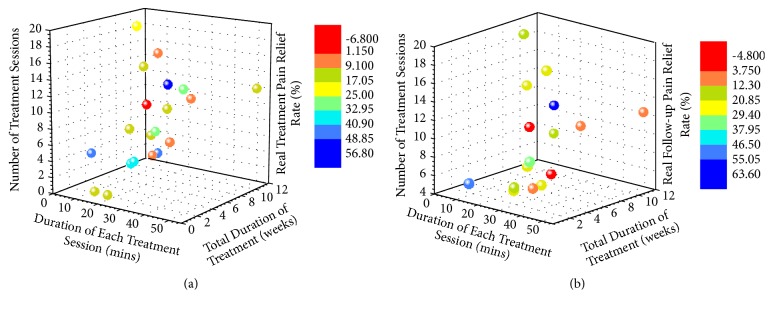
Three-dimensional distribution diagrams of three factors and pain relief rate. (a) Three factors (duration of each treatment session, total duration of treatment, and number of treatment sessions) and real treatment pain relief rate. (b) Three factors and real follow-up pain relief rate.

Three-dimensional distribution diagram of three factors and pain relief rate in both treatment and follow-up period was shown in Figure 6. In Figure 6(a), most of those 22 trials showed effective acupuncture; the pain relief percentage in 9 trials was beyond 20% (colored in green and blue), 15 trials above 10% (colored in yellow, green, and blue), 5 trials 1-10% (colored in orange), and 2 trials below 0% (colored in red). When the treatment schedule of two trials was the same, the points will be overlapped. From those 22 clinical trials, number of treatment sessions was set as 6-12 in 13 trials, total duration of treatment was costumed from 4 to 12 weeks in 18 trails, and 10 trials selected 20 mins and 7 trials chose 30 mins as the duration of each session.

In Figure 6(b), most of those 29 trials showed a long-term effective acupuncture; the pain relief percentage in 12 trials was beyond 20% (colored in bright yellow, green, and blue), 19 trials above 10% (colored in yellow, green, and blue), and 10 trials under 10% (colored in orange and red). When the treatment schedule of two trials is the same, the points will overlap.

## 4. Discussion

### 4.1. Strengths and Limitations

The review is a pilot to analyze randomized controlled trials of acupuncture treatment schedules for chronic pain; although Chinese trials and trials from Chinese databases are not assessed, specific acupuncture schedule that has been summarized with long-term clinical experience is usually used in the implementation of chronic pain randomized controlled trials conducted by Chinese doctors. Randomized controlled trials set by westerners who are not aware of the importance of acupuncture schedule were reviewed in this study. Moreover, the included studies are confined to randomized controlled trials, which greatly reduced selection bias. Limitations of our review mainly focus on the strong heterogeneity of the studies and the limited number of trials, as well as the distribution of parameters. For example, length of each session is mostly conducted for 20-30 mins. What is more, we focused on treatment schedule without in-depth exploration of details, such as point selection, depth of insertion, stimulation method, and needle retention time. Furthermore, we studied the three main components, D, N, and T, of “DOSE”, but other factors, e.g., deqi sensation, contributing to DOSE of acupuncture were not analyzed. However, linear regression coefficient is not a coefficient to measure causality. This data analysis should not be used directly as a basis for clinical treatment planning. Our primary aim is to draw attention to acupuncture schedules for protocol makers and detailed treatment protocols.

### 4.2. Interpretation of the Results

Firstly, the review implied that less frequency of acupuncture treatment can achieve the same effect just as that of higher frequency after treatment and less time in each session and less DOSE in weeks can achieve better follow-up outcome in patients with chronic pain. Our findings are highly consistent with some trials and analysis available in the literature. Zhang [38] reported that there was no difference between instant acupuncture and 30 min acupuncture in the contractile function of gallbladder. Shi [39] analyzed 350 cases of ophthalmic surgery performed with acupuncture anesthesia and found less time acupuncture was better than needle retention group. What is more, according to one of the earliest acupuncture books,* Huang Di Nei Jing Ling Shu [40]*, “the foot yang brilliance [conduit] is to be pierced 6 fen deep. [The needle] is to remain inserted for ten exhalations…The foot ceasing yin [qi conduits] is to be pierced 1 fen deep. [The needle] is to remain inserted for two exhalations.” It means the needle remains inserted less than 1 min. According to another classic book,* Zhen Jiu Jia Yi Jing [41]*, among all the 154 acupoints, there are only 15 points needles inserted for more than 10 exhalations. The longest needle retention time was the points gongsun, neiting, and huantiao, all of which were for 20 exhalations, about 1 min. Then in tang dynasty,* Qian Jin Fang [42]* wrote “a hundred exhalations between needles”; it means needle duration is about 5-6 mins. However, instant acupuncture was used by one of the included trials in the study. The number of instant acupuncture studies is too small to draw conclusions. If this result is confirmed, a lot of medical costs and social resources will be saved. Thus, further studies with more focus on duration of acupuncture session are suggested.

Secondly, our results show that the rate of pain relief dropped to the lower level after 18 weeks in the follow-up period. This suggests that the acupuncture treatment should be reconsidered at 18 weeks after needling to reinforce the effect. This new understanding could help to improve the long-term chronic pain control. The efficacy duration of acupuncture may be used as a criterion for efficacy evaluation in comparison with medicine and may provide new insight into mechanism research.

### 4.3. Implications for Future Research

We carefully evaluated the included trials and provided the following recommendations:

1. Standard treatment protocols should be conducted in future randomized controlled trials to ensure the effectiveness of acupuncture on chronic pain.
2. The recommendation treatment schedules should be added in STRICTA to evaluate the quality of performed randomized controlled trials.
3. High-quality randomized controlled trials are warranted. Randomization, patients blinding, evaluator blinding, acupuncturist experience, and needling sensation should be clearly described in randomized controlled trials.
4. Clinical effectiveness will be warranted if the time to receive acupuncture treatment and time when the retreatment is needed are clearly known.
5. The schedule related factors of acupuncture, such as frequency, DOSE, and total duration of treatment, should be taken into account when designing clinical trials of acupuncture.

## 5. Conclusions

Lack of agreement on the appropriate schedule of acupuncture treatment is an obstacle the improvement of clinical effectiveness of acupuncture and research into acupuncture. An effective acupuncture therapy clearly involves more than schedule. According to the results various factors related to acupuncture schedule influence the effect of chronic pain controlling. Each factor might have an effect on pain relief in a certain range, which should be considered in designing and interpreting clinical trials. The most reliable method to determine the appropriate acupuncture schedule is to compare different schedule parameters in a tightly controlled condition. We recommend that more high-quality randomized controlled trials about treatment schedule research should be carried out and more details of the reference range of specific parameters should be provided.

## Figures and Tables

**Table 1:** Characteristics of the included randomized controlled trials.

Author	Year	Country	Clinical problem	Score	Sample Size (persons)	Scale	Duration of Each Treatment Session (mins)	Total Duration of Treatment (weeks)	Number of Treatment Sessions	Frequency (sessions/week)	DOSE (mins/week)	Real Treatment Pain Relief	Length of Follow-up 1 (week)	Real Follow-up Pain Relief	Length of Follow-up 2 (week)	Real Follow-up Pain Relief	Length of Follow-up 3 (week)	Real Follow-up Pain Relief
Lewis [30]	2017	Ireland	Shoulder pain	5	227	SPADI	30	6	6	1.00	30	-3.17	24	3.05	48	0.54		
Karatay [13]	2017	Turkey	Fibromyalgia	5	75	VAS	30	4	8	2.00	60	29.74	4	38.50	12	36.04		
Ugurlu [12]	2017	Turkey	Fibromyalgia	4	50	VAS	30	8	12	1.50	45	32.24	(-)					
Naderinabi [26]	2017	Iran	Headache	4	150	VAS	unknown	12	30	2.50	unknown	15.34	(-)					
Garrido [31]	2016	Spain	Shoulder pain	5	68	VAS	20	4	4	1.00	20	37.25	12	26.66				
Vas [21]	2016	Spain	Fibromyalgia	5	164	VAS	20	9	9	1.00	20	16.33	15	8.37	39	17.68		
Hadianfard [29]	2015	Iran	Arm (Carpal Tunnel)	3	50	VAS	20	4	8	2.00	40	11.48	(-)					
Sahin [27]	2015	Turkey	Pelvis and hip	4	100	CPSI	20	6	6	1.00	20	16.25	2	18.27	10	19.17	18	25.83
MacPherson [23]	2015	UK	Neck Pain	3	517	NPQ	50	12	12	1.00	50	15.15	12	13.62	36	9.73		
Aranha [18]	2015	Brazil	Myofascial pain	4	60	VAS	30	4	8	2.00	60	-1.63	4	3.71				
Hinman [22]	2014	Australia	Knee Pain	4	282	WOMAC	20	12	9	0.75	15	5.07	36	11.10				
Itoh [34]	2014	Japan	Shoulder pain	6	18	VAS	30	5	5	1.00	30	37.50	5	30.07	15	25.59		
Chen [35]	2013	USA	Knee OA	6	214	WOMAC	20	9	12	1.33	27	56.78	14	63.57				
Tobbackx [28]	2013	Belgium	Neck (Whiplash)	4	39	VAS	20	1 day	1	1.00	20	10.82	(-)					
Weiss [24]	2013	Germany	Low Back Pain	3	143	SF-36	35	3	6	2.00	70	5.58	12	9.59				
Sozen [16]	2013	Turkey	Headache	3	56	VAS	20	5	20	4.00	80	20.65	12	18.48				
Hadianfard [19]	2012	Iran	Fibromyalgia	4	30	VAS	30	2	6	3.00	90	37.50	2	6.25	6	0.00	46	12.50
Itoh [36]	2012	Japan	Jaw	6	16	VAS	30	5	5	1.00	30	45.92	5	50.65				
Miller [17]	2011	Israel	Knee OA	3	55	KSS	20	8	16	2.00	40	-4.36	4	-25.27				
Molsberger [32]	2010	Germany	Shoulder pain	5	424	VAS	20	6	15	2.50	50	15.21	12	21.26				
Cherkin [37]	2009	USA	Low Back Pain	6	638	SBS	18	7	10	1.43	26	-6.78	19	-4.57	45	-4.61		
Lathia [33]	2009	USA	Headache (tension)	5	31	SPADI	30	6	12	2.00	60	no baseline	(-)					
Inoue [25]	2009	Japan	Low Back Pain	5	26	VAS	1	4	4	1.00	1	46.34	2	48.28	4	47.93		
Shen [20]	2009	Canada	Jaw	3	28	VAS	15	1 day	1	1.00	15	9.59	(-)					

(-): no follow-up; knee OA: knee osteoarthritis.

**Table 2:** Linear regression analysis on real treatment pain relief rate with schedule related factors.

Dependent Variable	Explanatory Variable	Coefficient Estimates of Predictors	R-square Value	P-value
Real Treatment Pain Relief Rate	Duration of Each Treatment Session	-0.187	0.010	0.655
	Total Duration of Treatment	-0.429	0.007	0.710
	DOSE	-0.051	0.005	0.765
	Frequency	-1.343	0.004	0.771
	Number of Treatment Sessions	-0.310	0.014	0.592
	Score	7.405	0.217	0.025

**Table 3:** Linear regression analysis on real follow-up pain relief rate with schedule related factors.

Dependent Variable	Explanatory Variable	Coefficient Estimates of Predictors	R-square Value	P-value
Real Follow-up Pain Relief Rate	Follow-up Weeks	-0.542	0.200	*0.015 *
	Duration of Each Treatment Session	-0.562	0.117	0.070
	Total Duration of Treatment	-0.504	0.007	0.672
	Number of Treatment Sessions	-0.475	0.011	0.584
	Frequency	-4.008	0.037	0.320
	DOSE	-0.220	0.105	0.086
	Score	5.219	0.093	0.108

## Supplementary Materials

Supplementary Materials Search Strategy in Pubmed, Embase, and Cochrane Central Register of Controlled Trials.
