# Supplementary material for: What Is the Appropriate Acupuncture Treatment Schedule for Chronic Pain? Review and Analysis of Randomized Controlled Trials
Source: Evid Based Complement Alternat Med. 2019 Jun 18;2019:5281039. doi: 10.1155/2019/5281039 (PMC6604345; doi:10.1155/2019/5281039)
Supplement: Supplementary Materials — Search Strategy in Pubmed, Embase, and Cochrane Central Register of Controlled Trials. [file 5281039.f1.pdf]

Search Strategy in Pubmed, Embase and Cochrane Central Register of Controlled Trials

Search Strategy in Pubmed

#1 Conditions: ((("chronic pain"[MeSH Terms])) OR (((((((((((((((((joint[Title/Abstract]) OR hand[Title/Abstract]) OR wrist[Title/Abstract]) OR shoulder[Title/Abstract]) OR back[Title/Abstract]) OR spine[Title/Abstract]) OR lumbar[Title/Abstract]) OR neck[Title/Abstract]) OR cervical[Title/Abstract]) OR hip[Title/Abstract]) OR knee[Title/Abstract]) OR arm[Title/Abstract]) OR leg[Title/Abstract]) OR limb[Title/Abstract]) OR jaw[Title/Abstract]) OR head[Title/Abstract]) OR pelvis [Title/Abstract])) OR (((((((((((((((((((((((((((((((((((rotator cuff tendinitis) OR ankylosing spondylitis) OR fibromyalgia) OR carpal tunnel syndrome) OR joint disorders) OR osteoarthritis) OR arthritis) OR rheumatoid arthritis) OR elbow pain) OR back pain) OR neck pain) OR neck disorder) OR shoulder pain) OR knee pain) OR tennis elbow) OR lateral elbow tendinopathy) OR subacromial bursitis) OR lateral epicondylitis) OR myofascial pain) OR heel pain) OR epicondyalgia) OR ankle distorsions) OR rheumatic disorders) OR tendinitis) OR rotator cuff lesions) OR tempromandibular pain) OR tempromandibular dysfunction) OR hemiplegic shoulder) OR contracture) OR knee extensor mechanism disorders) OR myalgia) OR arthralgia) OR gonarthrosis) OR extremity) OR extremities)

#2 Acupuncture: ((((((acupuncture[MeSH Terms]) OR "acupuncture therapy"[MeSH Terms]) OR "acupuncture points"[MeSH Terms]) OR body acupuncture) OR triggers point)

#3 Study Design: (((((((((((((((random[Title/Abstract]) OR random[MeSH Subheading]) OR randomized controlled trial[Publication Type]) OR double blind method) OR single blind method) OR placebos) OR clinical trial[Publication Type]) OR clinical trials) OR controlled clinical trial[Publication Type])) OR ((clin*[Title/Abstract]) AND trial*[Title/Abstract])) OR ((((((singl*[Title/Abstract]) OR doubl*[Title/Abstract]) OR trebl*[Title/Abstract]) OR tripl*[Title/Abstract])) AND ((blind*[Title/Abstract]) OR mask*[Title/Abstract]))) OR placebo*[Title/Abstract]) OR random*[Title/Abstract])) OR random[MeSH Terms]

#4: #1 AND #2 AND #3 Filters: Publication date from 2009/01/01 to 2018/12/31

#5: ((review[Publication Type]) OR pilot[Publication Type]) OR protocol[Publication Type] Filters: Publication date from 2009/01/01 to 2018/12/31

#6: #4 NOT #5

Total :1484

Search Strategy in Embase

('acupuncture':ab,ti OR 'acupoint':ab,ti OR 'needle':ab,ti) AND ('chronic pain':ab,ti OR 'bone pain':ab,ti OR 'headache':ab,ti OR 'limb pain':ab,ti OR 'musculoskeletal pain':ab,ti OR 'myalgia':ab,ti OR 'neuralgia':ab,ti OR 'pelvic pain':ab,ti) AND [randomized controlled trial]/lim AND [article]/lim AND [humans]/lim AND [2009-2018]/py

Total :183

Search Strategy in Cochrane Central Register of Controlled Trials

ID	Search	Hits
#2	MeSH descriptor: [Chronic Pain] explode all trees	1664

#3 (joint):ti,ab,kw OR (hand):ti,ab,kw OR (wrist):ti,ab,kw OR (shoulder):ti,ab,kw OR (back):ti,ab,kw (Word variations have been searched) 64839

#4 (spine):ti,ab,kw OR (lumbar):ti,ab,kw OR (neck):ti,ab,kw OR (cervical):ti,ab,kw OR (hip):ti,ab,kw (Word variations have been searched) 54100

#5 (knee):ti,ab,kw OR (arm):ti,ab,kw OR (leg):ti,ab,kw OR (limb):ti,ab,kw OR (jaw):ti,ab,kw (Word variations have been searched) 101224

#6 (head):ti,ab,kw OR (pelvis):ti,ab,kw (Word variations have been searched) 21707

#7 MeSH descriptor: [Acupuncture] explode all trees 140

#8 MeSH descriptor: [Acupuncture Therapy] explode all trees 4184

#9 MeSH descriptor: [Acupuncture Points] explode all trees 1764

#10 MeSH descriptor: [Randomized Controlled Trial] explode all trees 138

#11 (randomized controlled trial):pt OR (clinical trial):pt OR (controlled clinical trial):pt OR (double blind):pt OR (random):ti,ab,kw (Word variations have been searched) 563479

#12 #2 or #3 or #4 or #5 or #6 197781

#13 #7 or #8 or #9 4273

#14 #10 or #11 563617

#15 #12 and #13 and #14 with Cochrane Library publication date Between Jan 2009 and Dec 2018, in Trials 925
